# Autonomic Neuropathy and Albuminocytologic Dissociation in Cerebrospinal Fluid As the Presenting Features of Primary Amyloidosis: A Case Report

**DOI:** 10.3389/fneur.2017.00368

**Published:** 2017-07-26

**Authors:** Jingjing Li, Yi Li, Hongbing Chen, Shihui Xing, Huiyu Feng, Dawei Liu, Dilong Wang, Jinsheng Zeng, Yuhua Fan

**Affiliations:** ^1^Department of Neurology and Stroke Center, The First Affiliated Hospital, Sun Yat-sen University, Guangzhou, China; ^2^Department of Neurology, Guangzhou Red Cross Hospital, Medical College, Jinan University, Guangzhou, China; ^3^Department of Pathology, The First Affiliated Hospital, Sun Yat-sen University, Guangzhou, China

**Keywords:** autonomic neuropathy, albuminocytologic dissociation, primary amyloidosis, orthostatic hypotension, biopsy

## Abstract

**Objective:**

Primary amyloidosis is a disease with a poor prognosis and multi-organ involvement. Here, we report the clinical and pathological features of a patient with primary amyloidosis featuring autonomic neuropathy as the initial symptom and albuminocytologic dissociation in the cerebrospinal fluid (CSF).

**Methods:**

The patient was a 60-year-old Chinese male with numbness, orthostatic hypotension, and gastrointestinal symptoms. For diagnosis, we performed an electromyogram (EMG), lumbar puncture, Bence Jones protein urine test, serum electrophoresis blood test, sural nerve and rectal membrane biopsies, transthyretin (TTR) gene sequencing, and bone marrow puncture.

**Results:**

Congo red staining of sural nerve and rectal membrane biopsies showed amyloid deposition and apple-green birefringence was visualized under polarized light microscopy. TTR gene sequencing showed no causative mutation. Following lumbar puncture, normal CSF cell counts and elevated CSF protein concentration (1,680 mg/L) were detected. Bone marrow puncture showed that out of the total number of whole blood cells, 0.56% were abnormal plasma cells and that 87.4% of the total number of plasma cells were abnormal. EMG results showed mixed peripheral nerve damage predominately in the sensory nerve fibers.

**Conclusion:**

Obvious symptoms of neuropathy, particularly autonomic neuropathy, albuminocytologic dissociation, and organ function damage suggested a diagnosis of amyloidosis. In such patients, neurologists should use caution to differentiate between chronic inflammatory demyelinating polyneuropathy, primary amyloidosis, and familial amyloid neuropathy.

## Introduction

Amyloidosis is caused by the extracellular deposition of misfolded, insoluble proteins in organs and tissues, and is usually fatal when untreated (1). Neuropathies occur in approximately 35% of cases but are the presenting symptoms in 7.5% of cases (2, 3). However, less-invasive diagnostic tests, such as fat pad biopsy, can have low sensitivity (4). Amyloid deposits show characteristic yellow-green birefringence when viewed under polarized light with Congo red staining (1, 4). Neuropathy with prominent autonomic failure and end organ involvement point to a diagnosis of amyloidosis (5). Diarrhea, weight loss, and general autonomic failure are systemic hallmark features of amyloidosis that can be mistaken for diabetic neuropathy or chronic inflammatory demyelinating polyneuropathy (CIDP) (6). The causes of autonomic neuropathy are varied including diabetic autonomic neuropathy, amyloid neuropathy, acute and subacute autonomic neuropathies, immune-mediated and paraneoplastic neuropathies, hereditary autonomic neuropathies, and autonomic neuropathy due to infectious diseases and toxic (7). So the differential diagnosis is extremely important. As neurologists, we should pay attention to the systematic disease that influences the nervous system.

## Case Presentation

We analyzed a 60-year-old man who was admitted to our department complaining of a 1-year history of a loss of appetite with abdominal distension and an 8-month history of dizziness with numbness of the bilateral lower extremities. In addition, the patient had a history of type II diabetes mellitus for 14 years and hypertension for 25 years. The patient was diagnosed as diabetic peripheral neuropathy before he came to our hospital and the diagnosis on admission was (1) autonomic dysfunction; (2) type II diabetes mellitus; and (3) hypertension. Gradually, he suffered from aggravated dizziness when standing up from a squatting position. He also suffered from alternating bouts of diarrhea and constipation. We performed a physical examination, laboratory tests, brain magnetic resonance imaging (MRI), positron emission tomography/computed tomography (PET–CT), lumbar puncture, marrow puncture, and biopsy of the sural nerve and rectal membrane. The study protocol was approved by the local committee on human research, and the patient provided his informed consent.

### Clinical Findings

On examination, the patient’s blood pressure was 120/80 mmHg in the supine position and 60/40 mmHg in the standing position. He had normal consciousness and orientation. Muscle strength, tone of the extremities, and coordinate movement were all normal. There was an absence of sensation, bilaterally below the knee. All of the tendon reflexes were normal, with no pathological reflexes. Increased sweating from the chest was noted.

An electromyogram showed mixed peripheral nerve damage predominately in the sensory nerve fibers of the right extensor digitorum muscle and the tibialis anterior muscle. Doppler echocardiography showed diffuse hypertrophy of the left ventricular wall; the thickness of the ventricular septum was 17 mm. Plain and enhanced brain MRI scanning revealed mild brain atrophy in the bilateral hemisphere. Brain PET–CT was normal. Holter monitoring showed a second-degree atrioventricular block (type I, 2:1 block). The patient’s heart rate reduced to 30 beats per minute, and he was transferred to the Department of Cardiology for the implantation of a permanent pacemaker. A lumbar puncture was performed to collect cerebrospinal fluid (CSF); CSF analysis was normal, although CSF protein concentration (1,680 mg/L) was elevated. Transthyretin (TTR) gene sequencing showed no causative mutation. The Bence Jones protein urine test was positive, and the serum electrophoresis blood test was weakly positive for Bence Jones protein. Urinary protein or micro-albumin excretion was only mildly elevated.

### Pathological Results

**Figure 1:**
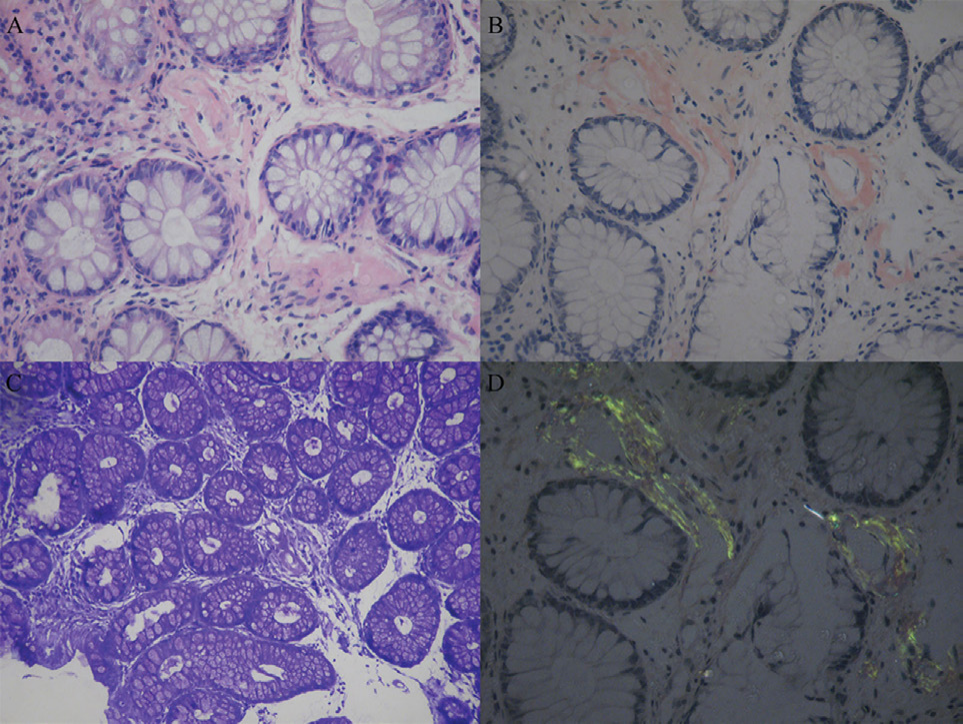
Amyloid deposition in the rectal membrane. Histopathological characteristics of amyloidosis involved the deposition of amyloid most commonly around blood vessels in the mesenchyme of the intestinal mucosa. **(A)** Hematoxylin and eosinstain. **(B)** Congo red specific stain. **(C)** Methyl violet stain. **(D)** Congo red stain under polarized light.

**Figure 2:**
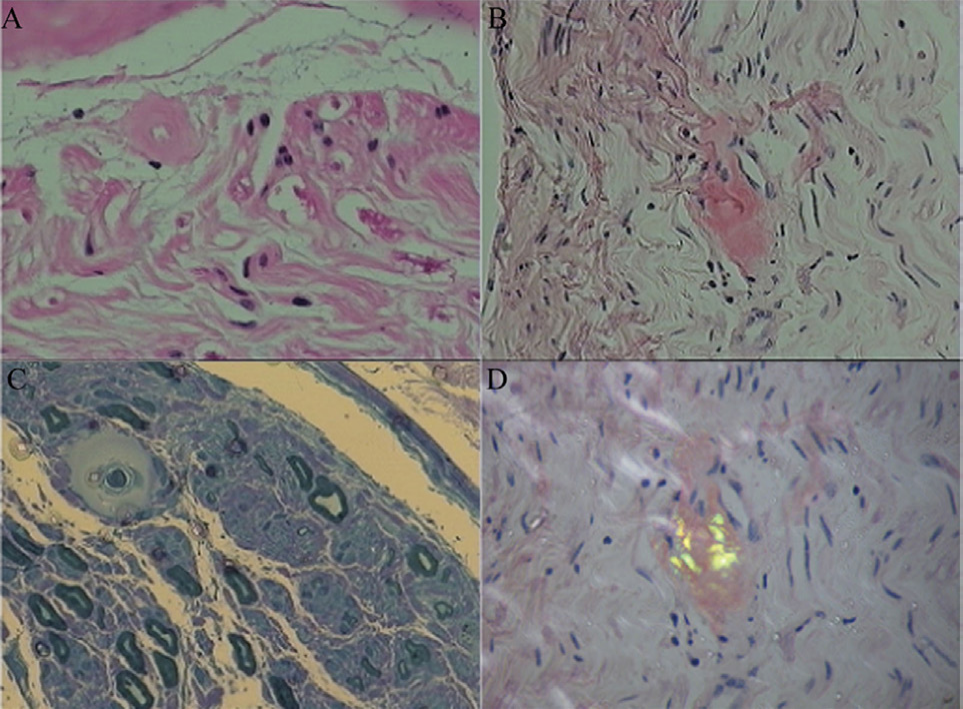
Amyloid deposition in the sural nerve. Histopathological characteristics showed endoneurial and perivascular amyloid deposition. **(A)** Hematoxylin and eosin stained cross-section of peripheral nerve demonstrating perivascular amyloid deposition. **(B)** Congo red specific stain without polarization. **(C)** Methyl violet stain. **(D)** Congo red staining of endoneurial amyloid with polarization.

Sural nerve and rectal membrane biopsies were performed. Congo red staining was positive, and apple-green birefringence was visualized under polarized light microscopy. Deposition of amyloid most commonly identified around blood vessels in the mesenchyme of the intestinal mucosa (Figure 1). Furthermore, endoneurial and perivascular amyloid deposition was detected in the sural nerve (Figure 2). Bone marrow smears showed active bone marrow hyperplasia, while bone marrow puncture indicated that the proportion of abnormal plasma cells were 0.56% of the whole blood cells and 87.4% of the total number of plasma cells.

### Treatment and Follow-up

The patient’s symptoms appeared to recover following gamma globulin implosive therapy (0.4 g/kg/day) and dexamethasone (10 mg/day) management. The difference in systolic blood pressure between the supine and standing position reduced to 20 mmHg, and the patient could walk for approximately 10 min without assistance. The frequency of diarrhea was reduced. However, his symptoms worsened 1 week after the improvement. He could sit for approximately 20 min, but he still felt dizzy when standing. In his follow-up visit 3 months after hospital discharge, the systolic blood pressure difference between the supine and standing position was approximately 30 mmHg, and he could walk for 200 m with a supportive device. He reported that he had experienced an episode of ventricular fibrillation during his sleep, in which he experienced reduced blood pressure and oxygen concentration, but his symptoms resolved after emergency treatment. In terms of his drug regimen, dexamethasone was replaced with hydrocortisone. The patient refused to be transferred to the Department of Hematology for treatment and went back to his home town. Unfortunately, the patient died in March 2017 due to ventricular fibrillation and renal failure. There was no autopsy result because the relatives of the patient refused to perform autopsy after the patient died.

## Discussion

Primary amyloidosis is a disease with a poor prognosis and multi-organ involvement. Diagnosis of this condition is difficult when the onset symptom is neuropathy, and the symptoms from other organs are uncertain. Patients with an apparent history of diabetes, who exhibit autonomic dysfunction such as orthostatic hypotension and gastrointestinal symptoms, could be easily misdiagnosed as having diabetic peripheral neuropathy or CIDP (8–10). The diagnosis of primary amyloidosis is usually ignored, particularly with significant albuminocytologic dissociation.

In a previous report, Maths et al. reported five cases of amyloidosis that showed symptoms similar to CIDP. In these five patients, CSF protein concentration was either mildly or significantly elevated (50–90 mg/dL). The symptoms of all five patients failed to improve following treatment with IVIg (2 g/kg body weight). Three of the five patients possessed an associated TTR gene mutation, and the other two patients were considered to have primary amyloidosis (8). Albuminocytologic dissociation can be detected if lesions locate in nerve roots or meningeal. The patient in the present study did not present with obvious positive signs of nerve root traction, and neither plain nor renhanced brain MRI scans showed signs of meningeal damage. However, we proved the existence of amyloidosis by analyzing the rectal membrane. Furthermore, in familial hereditary amyloidosis peripheral neuropathy, which is caused by a mutation of the TTR gene, the presentation can also be autonomic dysfunction. However, TTR negative results should not exclude the possible diagnosis of primary amyloidosis. Primary amyloidosis is characterized by clonal plasma cell disorder. The Bence Jones protein urine test, serum electrophoresis blood test, bone marrow puncture, and PET–CT are also useful in arriving at an accurate diagnosis.

The term autonomic neuropathy has often been used interchangeably with orthostatic hypotension, and gastrointestinal dysfunction has also been considered as a form of autonomic neuropathy (10). In our patient, we initially considered a diagnosis of orthostatic hypotension. We observed that the patient had a significantly widened ventricular septum, which is a positive sign of amyloid deposition (we did not perform cardiac biopsy due to being in possession of certain sural and rectal data). Since the autonomic nervous system controls the function of internal organs (7), it is therefore important that we ascertain whether symptoms are caused by autonomic neuropathy or damage to the organs.

We have demonstrated that a diagnosis of amyloidosis should not be ignored in patients presenting with neuropathic onset symptoms, particularly autonomic neuropathy (such as orthostatic hypotension and gastrointestinal symptoms). Amyloidosis is more likely to be confirmed in patients showing damage to other organs. The identification of albuminocytologic dissociation could also result in the misdiagnosis of CIDP. Biopsy therefore represents the golden standard for helping physicians to make an accurate diagnosis of amyloidosis. In addition, physicians should try to carefully differentiate between primary amyloidosis, familial amyloid neuropathy, and secondary amyloidosis. Early diagnosis can be helpful in treating patients effectively and thus increase the chances of survival. Our cases and recommendations are therefore highly meaningful for neurologists who are not familiar with primary amyloidosis.

## Conclusion

We recommend that neurologists pay very careful attention to amyloidosis in patients with onset symptoms involving peripheral neuropathy, particularly those with autonomic neuropathy damage and albuminocytologic dissociation. Early diagnosis will lead to more effective treatment regimens and increase the chances of patient survival.

## Ethics Statement

This study was carried out in accordance with the recommendations of ethic committee of First Affiliated Hospital of Sun Yat-Sen University with written informed consent from all subjects. All subjects gave written informed consent in accordance with the Declaration of Helsinki. The protocol was approved by the ethic committee of First Affiliated Hospital of Sun Yat-Sen University. The patient signed the consent form and gave consent to publish the case report when he was in our hospital. This study was carried out by all abovementioned authors. All participants gave written informed consent. They have authorized JL to submit or publish the work on their behalf.

## Author Contributions

JL and YF: design of the study, analysis of the data, and drafting the manuscript. JZ and HF: revising the manuscript for intellectual content. HC, SX, and DW: collecting data and analysis of data. DL and YL: interpretation of pathological data.

## Conflict of Interest Statement

The authors declare that the research was conducted in the absence of any commercial or financial relationships that could be construed as a potential conflict of interest.
